# AFP-HSP90 mediated MYC/MET activation promotes tumor progression in hepatocellular carcinoma and gastric cancers

**DOI:** 10.1186/s12935-024-03455-6

**Published:** 2024-08-12

**Authors:** Ziqi Lin, Rulu Pan, Liyue Wu, Fangsheng Zhu, Qiwei Fang, Hang Fai Kwok, Xincheng Lu

**Affiliations:** 1 School of Basic Medical Sciences, Wenzhou Medical University, Wenzhou, China; 2 Cancer Centre, Faculty of Health Sciences, University of Macau, Avenida da Universidade, Taipa, Macau SAR China; 3 MoE Frontiers Science Center for Precision Oncology, University of Macau, Avenida de Universidade, Taipa, Macau SAR China; 4 Department of Biomedical Sciences, Faculty of Health Sciences, University of Macau, Avenida da Universidade, Taipa, Macau SAR China

## Abstract

Alpha-fetoprotein (AFP) elevation is a well-known biomarker in various diseases, particularly in the diagnosis of hepatocellular carcinoma (HCC). Intracellular AFP has been previously implicated in promoting tumorigenesis. In this study, we discovered that AFP enhances the stability of oncoproteins c-MYC and c-MET, thereby facilitating the progression of liver and gastric tumors. Our findings suggest that AFP acts by stabilizing these oncoproteins, which are clients of heat shock protein 90 (HSP90), and prevents their degradation through ubiquitination. Intriguingly, we identified AFP as a novel co-chaperone of HSP90, demonstrating its ability to regulate the stabilization of HSP90 client proteins. Furthermore, our results indicate that inhibiting AFP or HSP90 enhances the cytotoxicity of chemotherapeutic agents in AFP-producing HCC and gastric cancer cells. These findings have significant implications for the development of therapeutic strategies targeting AFP-producing tumors, as the AFP-HSP90-mediated activation of c-MYC and c-MET provides new insights into potential treatment approaches. In summary, this study sheds light on the role of AFP in promoting tumor progression by stabilizing oncoproteins through its interaction with HSP90. The identification of this mechanism opens up new avenues for therapeutic interventions in AFP-producing tumors.

**Supplementary Information:**

The online version contains supplementary material available at 10.1186/s12935-024-03455-6.

## Introduction

Alpha-fetoprotein (AFP) is an important glycoprotein primarily produced during fetal development by the fetal liver and yolk sac. While AFP levels decrease postnatally, it plays diverse roles in biological processes, such as ligand transportation, chemotaxis, oxygen-free radical scavenging, and lipid peroxidation [1, 2]. In clinical contexts, AFP has been extensively studied in relation to developmental defects such as neural tube defects and brain/spinal cord malformations, and it is considered a biomarker for the diagnosis of chromosomal disorders [3, 4]. Additionally, AFP has been found to be essential for the hypothalamus-pituitary system in female mice, and its depletion can lead to anovulation and infertility in these mice [5, 6].

Elevated AFP levels in specific tumors, particularly hepatocellular carcinoma (HCC), have established it as a vital marker for cancer screening [7]. A strong correlation between AFP expression and high mortality rate was also found in HCC patients [8]. Beyond HCC, AFP production is observed in gastric cancer (GC), specifically AFP-producing gastric carcinoma (AFPGC), associated with higher metastatic risk and poorer prognosis [9, 10]. Research has extensively explored AFP in HCC, linking it to tumor growth through various mechanisms. Proposed mechanisms include AFP acting as a growth regulator in the bloodstream, impacting immune surveillance evasion and disrupting signaling pathways like Fas/FasL and TRAIL-induced cascades [11, 12]. Intracellularly, AFP participates in autophagy, apoptosis signaling, and tumor growth through processes like PI3K/Akt/mTOR pathway activation, Fas mRNA translation, and RAR nuclear translocation [13–17]. Despite these findings, understanding AFP's role in tumor progression and its underlying mechanisms remains incomplete.

A recent study revealed a correlation between elevated AFP and activated transcription factor c-Myc in HCC patients, prompting investigation into AFP's potential role in c-Myc regulation [18]. c-Myc, a well-known oncoprotein, is implicated in malignant transformation and tumor progression. Dysregulation of c-Myc is often associated with poor tumor prognosis [19–21]. High levels of c-Myc in human HCC and gastric cancer have been linked to an unfavorable prognosis, as it targets genes involved in crucial cellular functions such as cell growth, cell cycle regulation, apoptosis, and cellular transformation [20, 22, 23]. Similarly, increased c-Met expression was observed alongside enhanced AFP in AFP-producing tumors in our research. c-Met is a proto-oncogene product that is frequently amplified in various primary human tumors and is involved in regulating tumor progression, migration, and activation of downstream signaling pathways [24, 25]. Although elevated levels of c-Myc and c-Met have been reported in HCC and gastric cancer patients, it is not yet certain whether these proteins are responsible for AFP-promoted progression of liver and gastric cancers [10, 18]. Thus, this study aims to unravel the intricate regulation of c-Myc and c-Met by AFP, investigating their impact on tumor growth. By studying these regulatory mechanisms, we seek to enhance our understanding of AFP's contributions to tumor progression in liver and gastric cancers, providing insights into potential therapeutic strategies for AFP-producing tumors.

## Materials and methods

### Reagents and antibodies

Anti-Met (#3127), anti-CDK4 (#12790), anti-P21 (#27947), anti-Cyclin E1 (#20808), anti-DYKDDDDK Tag (#14793), anti-Myc-Tag (#2278), anti-Cytochrome c (#11940), anti-Bcl-2 (#15071) and anti-GAPDH (#2118) antibodies were purchased from Cell Signaling Technology. Anti-AFP (sc-166325), anti-Fas (sc-8009), anti-Cyclin D1 (sc-20044), anti-c-Myc (sc-40), anti-HSP90α/β (sc-13119), and anti-HA-Tag (sc-7392) antibodies were purchased from Santa Cruz Biotechnology. Sorafenib, Cisplatin and Ganetespib were purchased from Selleck (Selleck, China).

### Cell culture and transfections

Hepatocellular cancer cells (HLE) and gastric cancer cells (Fu97, SNU484) were purchased from the CoBioer Inc. (Nanjing, China). Hep3B cells were purchased from the Cell Bank of the Chinese Academy of Sciences (Shanghai, China). Expression vectors and siRNAs were introduced into cells by Lipofectamine 2000 (for vectors) or Lipofectamine RNAiMAX (for siRNAs) transfection reagents (Invitrogen, USA), respectively, according to the manufacturer’s instructions.

### Plasmids construction and siRNAs

pcDNA3.1-AFP plasmid was described previously [14]. Specific primers were used to clone the full-length AFP and MYC cDNA. The obtained products were subsequently subcloned into the p3xFLAG-CMV-10 vector (Sigma, E7658) and pcDNA 3.1/myc-his vector (Invitrogen, USA), respectively. The sequence of the plasmid was verified by standard sequencing. Pre-designed siRNAs were acquired from Genepharma (Shanghai, China). The sequences of siRNAs are described in Table S1.

### Lentiviral infection and stable cell lines construction

Lentivirus containing human AFP shRNA (shAFP 1#, 2#) or control shRNA (shNC) were purchased from GeneChem Co. (Shanghai, China). 72 h after infection, cells were selected in a medium containing puromycin (Santa Cruz, CA, USA) at the concentration of 2 μg/ml and then aspirated and replaced with a freshly prepared selective medium and culturing the surviving cells approximately every 2 days. For the selection of stably transfected cells, transfected SNU484 and HLE cells were grown in a medium containing 100 μg/ml G418 (Sigma, USA).

### MTT assay and colony formation assay

Cell viability was measured by MTT (Sigma, USA) assay as described in a previous study [14]. For colony formation assays, 150 shAFP-transfected Hep3B or Fu97 cells were seeded in 6-well plates and cultured for 10 days, with the medium replaced every 3 days. Thereafter, the colonies (≥ 50 cells per colony) were stained with 0.1% crystal violet (Sigma, USA). The experiments were conducted in triplicate and repeated three times.

### Quantitative RT-PCR

TRIzol Reagent (Invitrogen, USA) was used to extract total RNA from indicated cells, and a total of 2 μg of RNA was used for reverse transcription with MLV-reverse transcriptase kit (Invitrogen, USA). qPCR was carried out with SYBR Green (Tiangen, China) in triplicate assays on QuantStudio 6 Flex Real-Time System (Thermo, USA) according to the manufacturer’s instructions. The expression of mRNA was normalized to the expression levels of GAPDH and was calculated using the 2^−ΔΔCt^ method. The specific forward (F) and reverse (R) primer sequences were shown in Table S2.

### Dual-luciferase reporter assays

The human *c-Myc* 3′-UTR region (0.48 kb) was amplified from HEK293 cell genomic DNA and subcloned into a pGL3-control vector (Invitrogen, USA). 200 ng of pGL3 reporter plasmid containing target construct and 5 ng of Renilla luciferase vector (Invitrogen, USA) were co-transfected. Forty-eight hours after DNA transfection, the cells were harvested and analyzed using a dual-luciferase reporter assay system (Promega, USA). Firefly luciferase luminescence was measured and normalized to Renilla luciferase luminescence, then normalized to the control group accordingly. All groups were performed in triplicates.

### Flow cytometry analysis of cell cycle and apoptosis

For cell cycle assay, the indicated cells were transfected by AFP/control siRNA for 48 h. Cells were collected and permeabilized in 70% ethanol/PBS at − 20 °C for 12 h. After being centrifuged and washed with PBS, cells were then incubated with PI/RNase Staining Buffer (BD Biosciences, USA) for 15 min at RT. For apoptosis assay, cells were harvested 48 h after transfection and stained with an Annexin V-FITC/ propidium iodide (PI) Apoptosis Detection Kit (BD Biosciences, USA) in accordance with the manufacturer’s instructions. The cells were then analyzed on FACSCanto II flow cytometer (BD Biosciences, USA). All experiments were conducted in triplicate.

### Western blot analysis

Cells were lysed in lysis buffer supplemented with Protease/Phosphatase Inhibitor Cocktail (Cell Signaling Technology, USA), and protein quantification was performed as described previously [14]. Samples containing ~ 40 μg of proteins were separated using SDS–polyacrylamide gel electrophoresis and transferred to polyvinylidene difluoride membranes (Bio-Rad, USA). The membranes were blocked with 5% milk in PBST (0.1% Tween-20) and then incubated overnight at 4 °C with the appropriate dilutions of primary antibodies. After washing, the membranes were incubated with horseradish peroxidase (HRP)-conjugated secondary antibodies for 90 min. Protein bands were detected by the Immun-Star HRP Chemiluminescence kit (Bio-Rad, USA). ImageJ software was used to quantify the density of target bands.

### Ubiquitination assay

Indicated cells were transfected with the HA-ubiquitin plasmid. 24 h after transfection, the cells were treated with 25 μM proteasome inhibitor MG132 for 4 h and then lysed in ubiquitination assay buffer (50 mM Tris/HCl, pH 7.4, 150 mM NaCl, 1 mM EDTA and 1% NP40) containing Protease/Phosphatase Inhibitor Cocktail (Cell Signaling Technology, USA). Cell lysates were clarified and incubated with c-Myc or c-Met antibody overnight at 4 °C. The protein G-Sepharose beads (GE Healthcare, USA) were mixed with the whole-protein lysates and incubated for another 3 h at 4 °C. After being washed four times with ubiquitination wash buffer (50 mM Tris/HCl pH 7.4, 150 mM NaCl, 1 mM EDTA, 0.1% NP40), the beads were suspended with appropriate SDS loading buffer, and boiled for 10 min. Western blot was performed with HA-tagged antibody to detect ubiquitinated protein.

### Co-immunoprecipitation

Co-immunoprecipitation was performed as described in a previous study. Briefly, cells were lysed in an immunoprecipitation assay buffer (50 mM Tris/HCl, pH 7.5, 150 mM NaCl, 1% NP-40, and 1 mM EDTA) supplemented with the Protease/Phosphatase Inhibitor Cocktail (Cell Signaling Technology, USA). Following centrifugation at 12,000 rpm for 15 min to remove the debris, the cell lysates were incubated with an AFP (sc-166325, Santa Cruz, USA) or HSP90 α/β (sc-13119, Santa Cruz, USA) antibody at 4 °C overnight, and an IgG antibody was used as a negative control. The protein G-Sepharose beads (GE Healthcare, USA) were mixed with the whole-protein lysates and incubated for another 3 h at 4 °C. After being washed four times with the immunoprecipitation assay buffer, the beads were suspended with appropriate SDS loading buffer, and boiled for 10 min. Western blot was used to analyze protein expression.

### Immunofluorescence imaging

Immunofluorescence analysis was performed as described in a previous study. Briefly, Fu97 and Hep3B cells were grown on coverslips for 24 h and fixed in 4% formaldehyde for 20 min at RT and permeabilized with 0.1% Triton X-100/PBS for 10 min at 4 °C. After blocking with 1% BSA/PBS for 1 h at RT. The cells were then incubated overnight with anti-DYKDDDDK Tag (1:100 dilution; #14793, Cell Signaling Technology, USA) and HSP90α/β (1:100 dilution; sc-13119, Santa Cruz, USA) at 4 °C. The cells were washed three times in PBS and then incubated with fluorophore-conjugated secondary antibodies for 2 h at RT. The coverslips were mounted on glass slides in the presence of DAPI for nuclear staining, and cell images were recorded using a Nikon confocal microscope.

### Statistical analysis

Microsoft Excel and GraphPad Prism were used to analyze results including quantitative RT-PCR, MTT assay and colony formation assay. Parametric data between paired groups were analyzed using a t-test, and multigroup comparisons were performed using a Chi-square test. Data are presented as mean ± standard deviation (SD). Kaplan–Meier curve was used to evaluate the survival data with the log- rank test by GraphPad Prism software. p value < 0.05 was considered statistically significant. Cox’s proportional hazards model was used for multivariate survival analysis.

## Results

### Intracellular AFP promotes HCC and gastric cancer progression

**Fig. 1:**
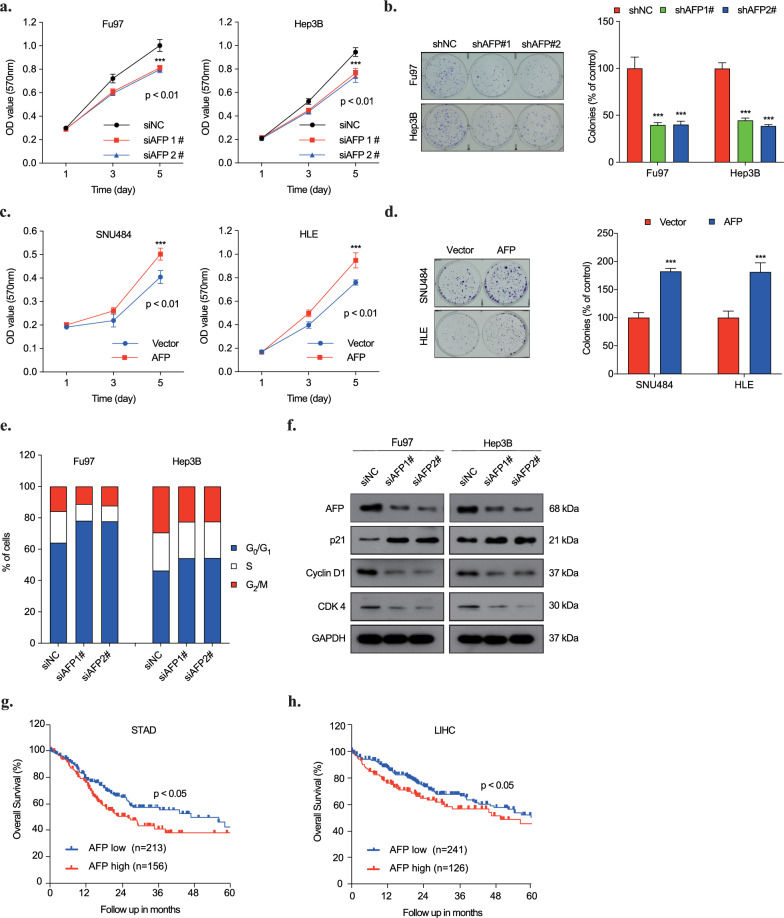
The oncogenic role of intracellular AFP in gastric and liver cancers. **a, b** Cell proliferation curve (**a**) and colony formation assays (**b**) of Fu97 and Hep3B cell lines with or without AFP knockdown.***p < 0.01 vs siNC/shNC. **c, d** Cell proliferation curve (**c**) and colony formation assays (**d**) of SNU484 and HLE cell lines with and without AFP overexpression. ***p < 0.01 vs Vector. **e** Fluorescence-activated cell sorting analysis (FACS) was performed on Fu97 and Hep3B cells stably expressing shAFP/control shRNAs. The percentage of cells in each cycle phase was calculated. ***p < 0.01 and *p < 0.05 vs siNC. **f** Western blot analysis of indicated cells transfected with negative control or AFP siRNAs. Cell lysates were immunoblotted and analyzed with P21, Cyclin D1, Cyclin E1 and CDK4 antibodies. **g, h** Kaplan–Meier plots comparing OS of HCC (**g**) and GC (**h**) patients according to the AFP mRNA abundance from TCGA dataset

Building upon our previous study focused on AFP's cellular machinery in HCC cells and DEN-induced liver tumors in mice [14], we expanded our investigation into the role of AFP in gastric cancer. Using two distinct short-interfering RNAs (siAFP 1#,2#), we knocked down AFP expression in the AFP-producing gastric cancer cell line, Fu97, and liver cancer cell line, Hep3B. The MTT assay and in vitro colony formation assay results recealed a significant suppression of proliferation in both AFP-producing tumor cell lines upon AFP knockdown (Fig. 1a, b; Fig. S1a, S1b). Conversely, overexpression of AFP in non-AFP producing cells, SNU484 and HLE, led to a notable promotion of cell proliferation (Fig. 1c, d; Fig. S1c, S1d). Analysis of apoptosis and cell cycle distribution showed increased apoptosis in Fu97 and Hep3B cells following AFP knockdown, with changes in the expression of apoptosis-associated proteins (Bcl-2 and cytochrome c), suggesting activation of the intrinsic apoptotic pathway (Fig. S1e, S1f). These findings mirrored those observed in HCC, indicating that AFP knockdown could activate the apoptosis pathway in gastric cancer. (Fig. S1f, S1g). AFP knockdown also induced cell cycle arrest at G_0_/G_1_ phase (Fig. 1e), accompanied by decreased expression of cell cycle regulators CyclinD1 and CDK4, and an increase in cyclin-dependent kinase inhibitor, P21^Waf1/Cip1^(Fig. 1f). Furthermore, exploring the associations between AFP expression and prognosis in liver and gastric cancer patients, we found that higher level of AFP expression had a shorter overall survival time compared to those with a lower level of AFP expression (Fig. 1g, h). These emphasize the role of intracellular AFP in driving cancer progression and suggests its potential as a prognostic marker for liver and gastric cancer patients.

### AFP promotes liver and gastric cancer growth by elevating c-Myc and c-Met expression

**Fig. 2:**
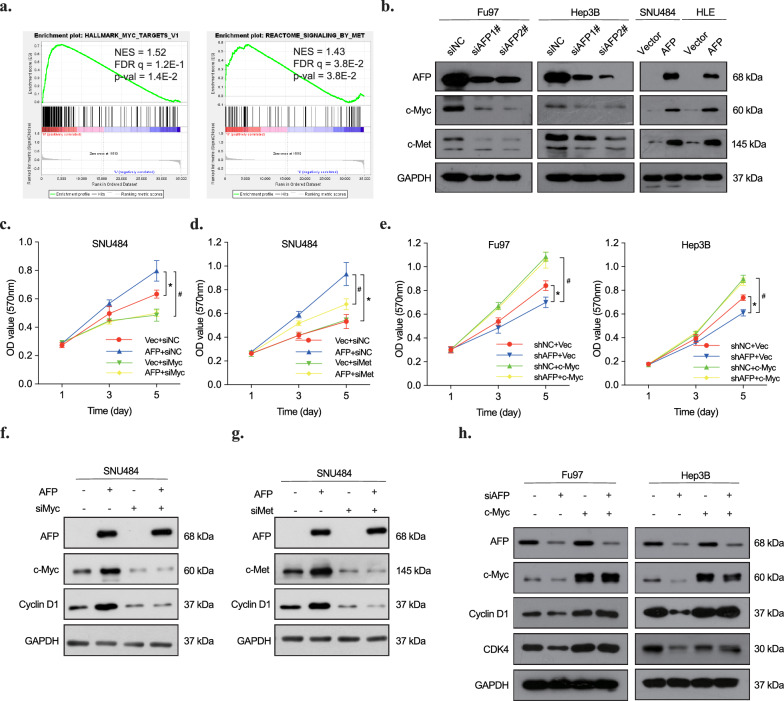
AFP promotes gastric and liver cancer growth through c-Met and c-Myc elevation. **a** Gene set enrichment analysis on liver cancer tissues from the public database (GSE36376). **b** c-Myc and c-Met protein level in AFP siRNA transfected Fu97 and Hep3B cells, as well as AFP plasmid transfected SNU484 and HLE cells. **c** Cell proliferation assay of SNU484 cells transfected with control plasmid, AFP plasmid, c-Myc siRNA, c-Myc siRNA and AFP plasmid (n = 5). *p < 0.05 vs Vec + siNC, #P < 0.05 vs AFP + siMyc. **d** Cell proliferation assay of SNU484 cells transfected with control plasmid, AFP plasmid, c-Met siRNA, c-Met siRNA and AFP plasmid (n = 5). *p < 0.05 vs Vec + siNC, #p < 0.05 vs AFP + siMet. **e** Cell proliferation assay of Fu97 (**e**) and Hep3B (f) cells transfected with control siRNA, AFP siRNA, c-Myc plasmid, AFP siRNA and c-Myc plasmid (n = 5). *p < 0.05 vs siNC + Vec, #P < 0.05 vs siAFP + c-Myc. **f** The expression of indicated proteins were detected by western blot in SNU484 cells transfected with AFP plasmid and c-Myc siRNAs or negative control. **g** The expression of indicated proteins were detected by western blot in SNU484 cells transfected with AFP or c-Met siRNAs or negative control. **h** The expression of indicated proteins were detected by western blot in Fu97 and Hep3B cells transfected with AFP siRNAs and c-Myc plasmid or negative control

To investigate the underlying mechanism, we referred to a study by Parpart et al., revealing transcription factor c-Myc activation in HCC patients with high AFP levels [18], indicating a potential role of c-Myc in AFP-expressing tumors. To confirm this, we analyzed the HCC cohort dataset (GSE36376) using GSEA analysis to compare the gene expression between patients with high and low AFP expression levels. The enrichment analysis demonstrated up-regulated gene sets in the high AFP expression group (Fig. S2a). The analysis revealed that the c-Myc target gene set (HALLMARK MYC TARGETS V1) was positively related to AFP expression, along with the c-Met signaling pathway (Fig. 2a). The positive correlation between c-Myc and AFP, and that between c-Met and AFP, was further supported by our experiments, where we knocked down or overexpressed AFP in various cell lines (Fig. 2b). To determine whether c-Myc and c-Met upregulation mediates AFP-induced cell proliferation and cell cycle progression, we inhibited c-Myc and c-Met expression respectively in SNU484 cells transfected with AFP plasmid. Our results showed that AFP expression promoted the proliferation of SNU484 cells, and the knockdown of c-Myc completely reversed the proliferation effect caused by AFP overexpression (Fig. 2c). While c-Met knockdown partially impaired the AFP-induced cell proliferation in HLE and SNU484 cells (Fig. 2d, Fig. S2b). These findings indicate the requirement of c-Myc and c-Met upregulation for AFP-promoted cancer cell growth. Further observations revealed that increased c-Myc expression enhanced the shAFP-mediated proliferation inhibition in Fu97 and Hep3B cells (Fig. 2e, f). Silencing either c-Myc or c-Met expression in AFP-expressing cells partially attenuated the expression of Cyclin D1 in SNU484 cells (Fig. 2g, h). Similar effect was also observed in in HLE cells with c-Met knockdown (Fig. S2c). Conversely, excess c-Myc reversed the inhibitory effects of AFP depletion on down-regulating Cyclin D1 and CDK4 (Fig. 2h). In summary, these results underscore the essential role of c-Myc and c-Met upregulation in AFP-mediated cancer cell growth, influencing proliferation and cell cycle promotion in AFP-producing liver and gastric cancer cells.

### Loss of AFP induces the ubiquitination and degradation of c-Myc and c-Met

**Fig. 3:**
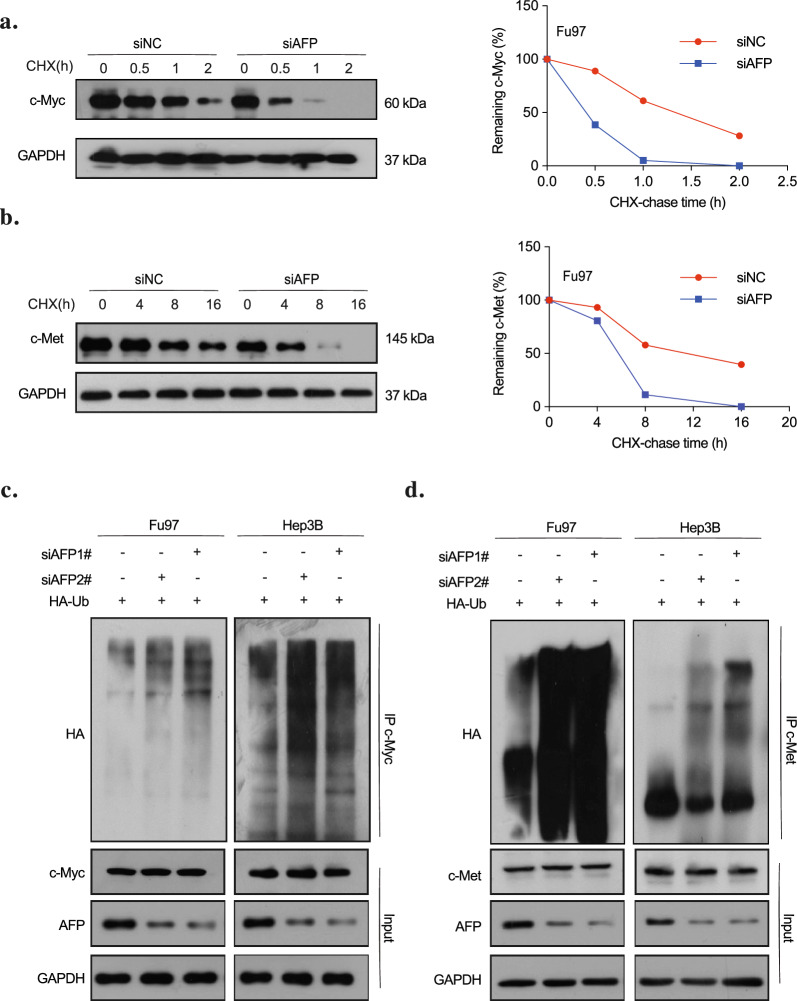
AFP inhibits c-Met and c-Myc ubiquitin–proteasome degradation. **a**, **b** Cycloheximide (CHX)-chase assay. Fu97 cells were transfected with siAFP or negative control as indicated, and then treated with cycloheximide (100 μg/ml) and harvested at indicated time points. The c-Myc (**a**) and c-Met (**b**) proteins were detected by western blotting (left panel). The graph shows quantification of relative c-Myc and c-Met levels (comparing to initial timepoint, right panel). (**c,d**) Ubiquitination of c-Myc and c-Met in Hep3B and Fu97 cells with AFP knockdown. Cells were transfected with AFP siRNAs (#1 and #2) or negative control as well as HA-tagged Ub plasmid, and then treated with 25 μM MG132. The cells were then harvested for the ubiquitylation assay

To understand how AFP promotes their expression, we explored processes regulating c-Myc and c-Met, including amplification, translocation, mRNA stabilization, and protein stabilization [26]. Surprisingly, qRT-PCR results in liver and gastric cancer cells transfected with AFP siRNA or plasmids showed no impact on their transcription levels (Fig. S3a), despite a positive correlation between AFP protein expression and that of c-Myc and c-Met in western blot data. Although c-Myc expression was also reported to be regulated through its mRNA 3’UTR [27], further investigating c-Myc translation through dual-luciferase reporter assay revealed no influence of AFP, suggesting that AFP does not affect the translational efficiency of c-Myc (Fig. S3b). Then a cycloheximide (CHX) chase assay was conducted to tracked the protein degradation rate of c-Myc and c-Met, and the results demonstrated that AFP depletion significantly impaired the half-life of endogenous c-Myc and c-Met, indicating a positive role of AFP in stabilizing c-Myc and c-Met (Fig. 3a,b). Moreover, we found that the ubiquitylation of c-Myc and c-Met were upregulated upon the downregulation of AFP in Fu97 and Hep3B cells, indicating that AFP reduces their levels of ubiquitylation (Fig. 3c,d). This relationship was confirmed by overexpressing AFP in SNU484 cells (Fig. S3c). Taken together, these results suggest that AFP stabilizes c-Myc and c-Met post-translationally by reducing their levels of ubiquitylation, which may confer their tumorigenic properties through corresponding pathways.

### AFP induces c-Myc and c-Met stabilization in an HSP90-dependent manner

**Fig. 4:**
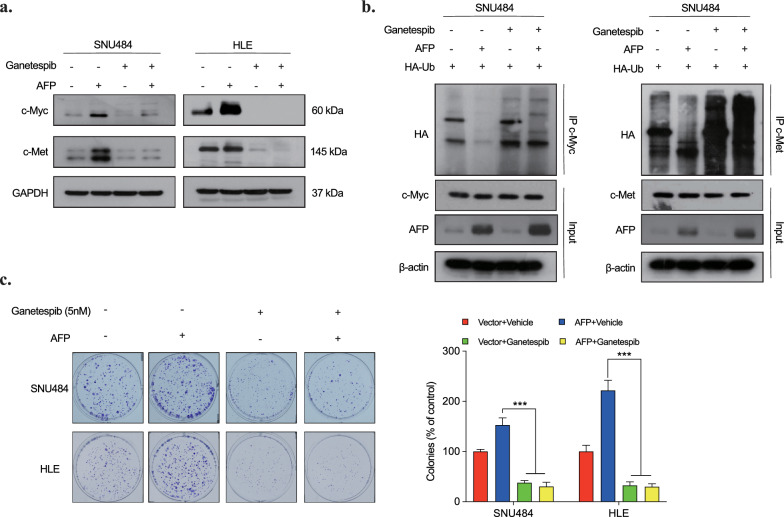
HSP90 enhances the stability of c-Met and c-Myc in AFP-producing cells. **a** Effect of Ganetespib treatment on endogenous c-Myc and c-Met in AFP-overexpressing cells. **b** Poly-ubiquitination level of c-Myc and c-Met by Ganetespib treatment in AFP- overexpressing cells. **c** Colony formation of SNU484 and HLE (with/without AFP overexpression) under Ganetespib treatment. Experiments were performed in triplicates. *p < 0.05 vs Vector + Vehicle, ^#^p < 0.05 vs AFP + Ganetespib

The known interaction between c-Myc and c-Met with heat shock protein 90 (HSP90) and the destabilizing effect of HSP90 inhibition on these proteins led us to investigate a common underlying mechanism by which AFP influences their expression [28, 29]. Blocking the ATP-binding site of HSP90 with Ganetespib, a HSP90 inhibitors, we examined the ubiquitinylation of c-Myc and c-Met in AFP-overexpressing SNU484 and HLE cells cells. Results demonstrated that Ganetespib treatment abrogated AFP-mediated upregulation of c-Myc and c-Met (Fig. 4a), restoring their poly-ubiquitination levels (Fig. 4b). Consistently, HSP90 inhibition significantly reduced the AFP-induced proliferation in SNU484 and HLE cells, as shown by the colony formation assay (Fig. 4c). These findings collectively suggest that AFP's impact on c-Myc and c-Met involves the inhibition of their ubiquitination through HSP90. Consequently, this process leads to the degradation of c-Myc and c-Met, ultimately suppressing tumor growth.

### AFP interacts with HSP90 and regulates the stability of c-Met and c-Myc

**Fig. 5:**
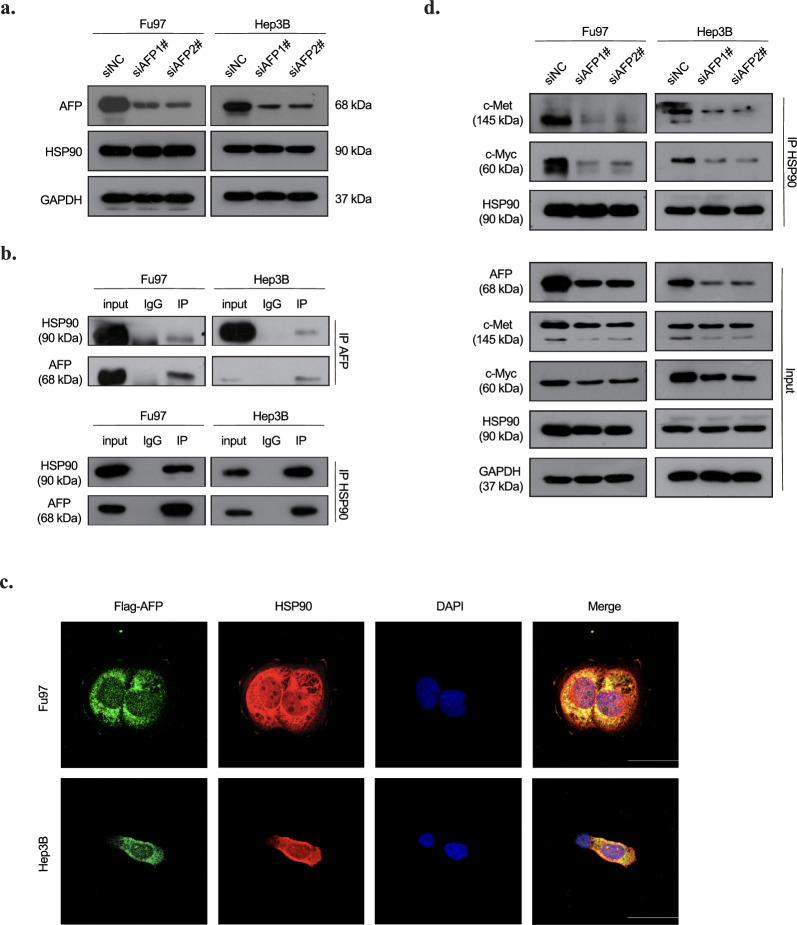
AFP-HSP90 interaction enhances the HSP90 binding with c-Met and c-Myc. **a** HSP90 expression level in AFP siRNA transfected Fu97 and Hep3B cells. **b** The interaction of AFP and HSP90 in the Fu97 and Hep3B cells. Fu97 and Hep3B cells whole-cell lysates were immunoprecipitated with control IgG, anti-AFP or anti-HSP90 antibody, followed by western blot with indicated antibodies. **c** Colocalization of AFP and HSP90 in Fu97 and Hep3B cells. scale bar, 20 μm. **d** Immunoprecipitation of HSP90 with c-Myc and c-Met in indicated Fu97 and Hep3B cells

Interestingly, neither the downregulation nor overexpression of AFP affected the expression of HSP90 in liver and gastric cancer cells, as confirmed by immunoblotting (Fig. 5a). Co-immunoprecipitation experiments using antibodies against AFP or HSP90 verified their interaction (Fig. 5b). And immunostaining revealed colocalization of AFP and HSP90 in the cytoplasm. (Fig. 5c). Considering the essential role of HSP90-client complexes in maintaining client protein stability [30], we investigated whether the interactions between HSP90 and c-Met or c-Myc were affected by AFP expression manipulation. Results demonstrated a significant decrease in the abundance of HSP90-associated c-Myc or c-Met following AFP knockdown in Hep3B and Fu97 cells (Fig. 5d). These findings indicate that AFP potentially strengthens the interaction between HSP90 and c-Myc or c-Met, leading to the stabilization of these oncogenic proteins. In summary, our results elucidate the mechanism by which AFP contributes to tumor growth by modulating the HSP90-client complex. The intricate interplay between AFP and HSP90, impacting the stability of critical oncogenic proteins, sheds light on potential therapeutic targets for AFP-associated cancers.

### Upregulation of AFP may confer chemodrugs resistance by activating c-Myc/c-Met signaling

**Fig. 6:**
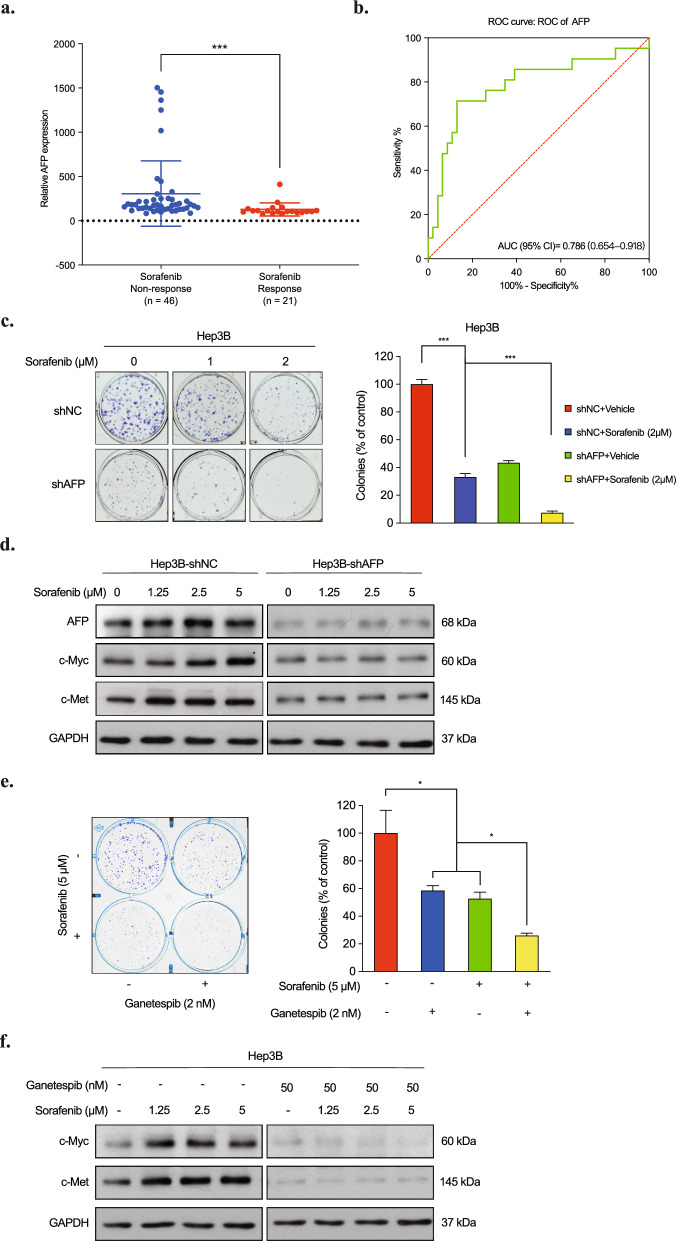
AFP confers sorafenib resistance on HCC cells by activating c-Myc/c-Met signaling. **a** Relative AFP expression comparison of responsive and non-responsive patients for sorafenib treatment (GSE109211). **b** The ROC curve analysis of AFP for sorafenib sensitivity in HCC patients (GSE109211). **c** Colony formation assays of AFP knockdown Hep3B cells treated with sorafenib. **d** AFP, c-Myc and c-Met level in AFP knockdown Hep3B (Hep3B-shAFP) and control cells (Hep3B-shNC) treated with indicated concentration of sorafenib. **e** Combination treatment of Ganetespib and Sorafenib synergistically inhibited Hep3B cell growth. **f** c-Myc and c-Met level in Hep3B cells treated with indicated concentration of sorafenib and ganetespib

Sorafenib, the standard systemic treatment for advanced hepatocellular carcinoma (HCC), faces limited efficacy, and identifying predictive biomarkers for sorafenib response is crucial [31, 32]. Recent studies propose AFP as a valuable biomarker for discriminating sorafenib-responsive and non-responsive patients. In a sorafenib-treated liver cancer cohort, differences in AFP expression between responsive and non-responsive groups were observed, suggesting AFP's potential in predicting sorafenib response (Fig. 6a). ROC curve analysis with an AUC of 0.786 supported AFP's potential as a biomarker for discriminating sorafenib-resistant patients (Fig. 6b). Further investigation into the role of AFP in HCC suggested the AFP-Myc/Met axis potentially contribute to sorafenib resistance. Increased sensitivity to sorafenib was observed in AFP-silenced Hep3B cells (Fig. 6c), and sorafenib-treated cells displayed elevated c-Myc and c-Met expressions, countered by AFP interference (Fig. 6d). Given its prominent application in HCC treatment, combination therapies involving sorafenib and other agents have been explored [32]. Combining sorafenib with the HSP90 inhibitor ganetespib, an AFP-Myc/Met axis inhibitor, resulted in enhanced inhibition of Hep3B colony formation (Fig. 6e). Western blot analysis revealed that ganetespib reversed c-Myc and c-Met expression induced by sorafenib, supporting the role of the AFP-Myc/Met axis (Fig. 6f). These results indicated that the combination of sorafenib and the HSP90 inhibitor ganetespib may have a synergistic inhibiting effect on AFP-expressing HCC cells, and therefore may be a potential treatment option for HCC patients.

The relationship between AFP expression and cisplatin efficacy in gastric cancer cells was also explored. AFP-silenced gastric cancer cells exhibited significantly stronger cisplatin sensitivity (Fig. S4a). Elevated c-Myc and c-Met in cisplatin-treated Fu97 cells were mitigated by AFP-silencing or ganetespib treatment (Fig. S4b,c). These results suggested the potential importance of AFP as a predictive biomarker for cisplatin response in gastric cancer and suggest that AFP-silencing may enhance the efficacy of cisplatin-based chemotherapy in AFP-expressing gastric cancer patients. Further studies are needed to validate these findings and unravel the underlying mechanisms, highlighting the potential of AFP as a predictive and therapeutic target in both HCC and gastric cancer.

## Discussion

Alpha-fetoprotein (AFP), a member of the albumin superfamily, is highly abundant in human fetal serum and serves as a tumor-associated biomarker in various cancers, including hepatocellular carcinoma (HCC) and gastric carcinoma. High AFP levels are associated with poor prognosis and high mortality rates in cancer patients [8]. This study explored the crucial role of AFP in HCC and gastric carcinoma, uncovering its involvement in cancer proliferation through the regulation of c-Myc and c-Met. Knockdown of AFP results in inhibition of Cyclin D1/CDK4 expression, and G1 arrest of cell cycle. c-Myc and c-Met, known regulators in tumor development, are effected by AFP. c-Myc deregulation is known to play a role in uncontrolled cell proliferation and is frequently involved in the initiation and development of various tumors [33]. c-Met, on the other hand, is a tyrosine kinase receptor that contributes to oncogenesis and tumor progression while regulating the migration of cancer cells [24]. Our study revealed that AFP stabilized c-Myc and c-Met by inhibiting their ubiquitination-mediated degradation, thereby promoting their oncogenic effects.

**Fig. 7:**
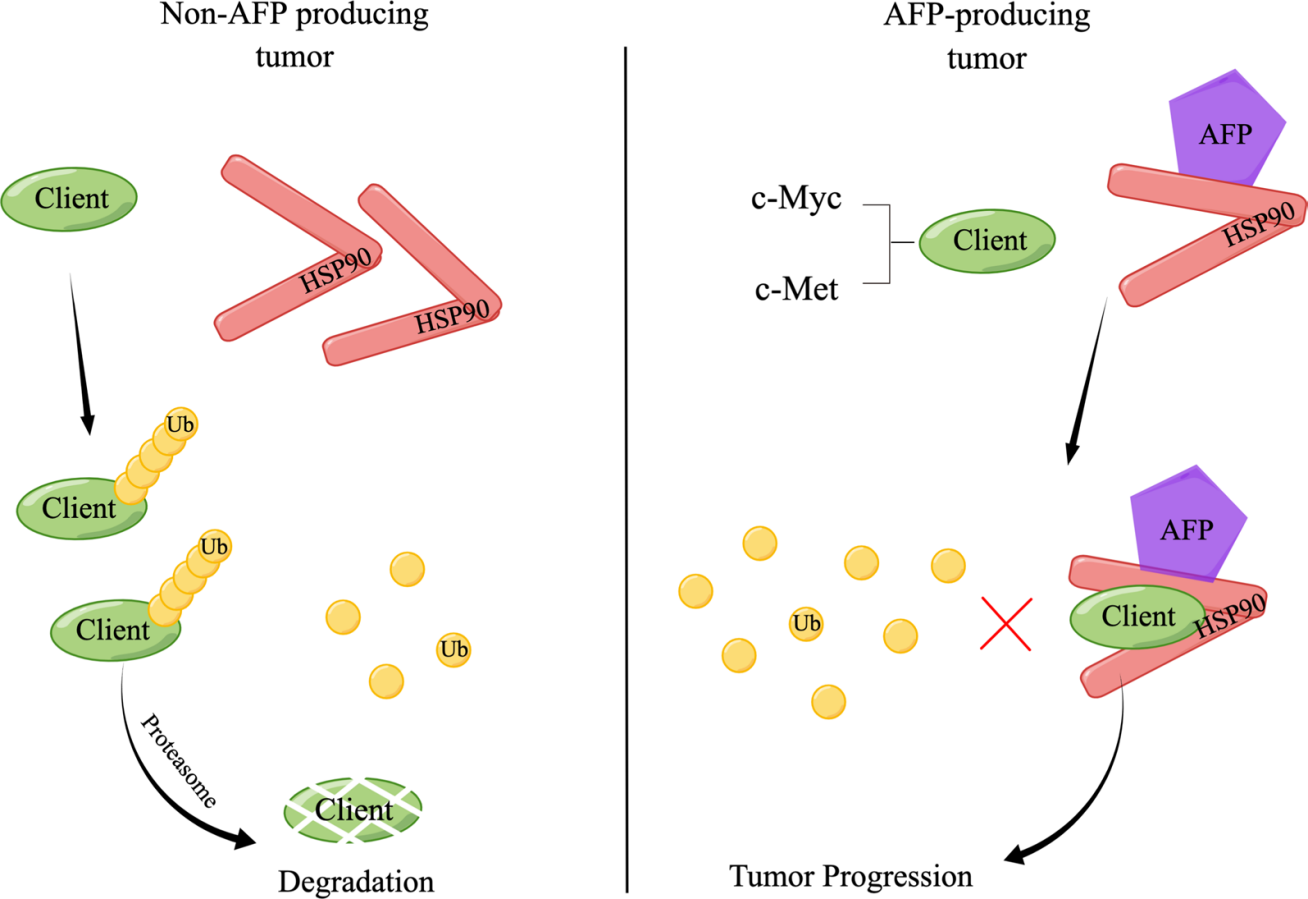
Schematic of proposed model illustrating AFP regulates tumor progression through* stabilizing c-Myc and c-Met.* AFP interacts with HSP90 chaperon and promotes the binding of HSP90 to c-Myc or c-Met, which in turn increases the stability of c-Myc and c-Met through inhibiting their ubiquitination-mediated degradation

The study also implicated heat shock protein 90 (HSP90) in AFP-mediated regulation of c-Myc and c-Met degradation. HSP90 is a molecular chaperone that plays important roles in cell proliferation, cell cycle progression, apoptosis, and drug resistance [34–36]. It has been reported to stabilize several oncogenic proteins, and inhibiting HSP90 has been shown to result in the misfolding and degradation of oncoproteins, thereby suppressing malignant transformation in various cancers, including HCC and gastric cancer [37, 38]. Increased expression of HSP90 has been associated with larger tumor size, higher tumor grade, and advanced stage of HCC [36]. HSP90 has also been implicated in promoting the growth and survival of HCC cells, making it a potential therapeutic target [39]. Additionally, elevated levels of plasma HSP90 have been considered as a potential biomarker for GC and HCC diagnosis [40, 41]. Our study found that HSP90 is essential for AFP-mediated enhancement of c-Met and c-Myc, and these proteins were shown to interact with HSP90 [42]. HSP90 inhibitors, such as Ganetespib, bind to the N-terminal ATP-binding site of HSP90, thereby suppressing ATP hydrolysis and the protein folding process. This leads to the degradation of client proteins primarily through the ubiquitin–proteasome pathway [43]. Consistent with these findings, we demonstrated that inhibiting HSP90 with Ganetespib increases the degradation of c-Myc and c-Met through the ubiquitin–proteasome pathway, resulting in the suppression of cell proliferation in AFP-expressing cells. Furthermore, our findings showed that AFP enhances the interaction between HSP90 and c-Myc/c-Met, contributing to the instability of c-Myc and c-Met through ubiquitination-mediated degradation. Through co-immunoprecipitation and immunofluorescent staining, we identified AFP as a previously unknown interacting partner of HSP90. Based on these results, we propose an AFP-HSP90-Myc/Met axis, where AFP interacts with HSP90 and promotes the binding of HSP90 to c-Myc or c-Met. This interaction increases the stability of c-Myc and c-Met by inhibiting their ubiquitination-mediated degradation (Fig. 7). The AFP-HSP90-Myc/Met axis may play a crucial role in the growth of AFP-producing tumors through the oncogenic effects of c-Myc and c-Met.Importantly, our study provides evidence that the AFP-HSP90-Myc/Met axis may be a potential pathway related to chemoresistance in HCC. Sorafenib, a multi-kinase inhibitor, is the first-line chemotherapy for advanced HCC patients, but its effectiveness is limited to a specific range of patient populations [32]. Preclinical and clinical studies have reported that AFP-producing HCC shows poor response to sorafenib [44]. This suggests that there may be crucial differences in the efficacy of sorafenib based on AFP expression levels, and the underlying mechanisms are not yet clear. Studies have indicated that upregulation of c-Myc and c-Met is a crucial mechanism for acquired resistance of hepatocellular carcinoma cells to sorafenib [45–48]. Our study demonstrated that knockdown of AFP restored the susceptibility of HCC cells to sorafenib treatment. Furthermore, either AFP knockdown or treatment with an HSP90 inhibitor attenuated the sorafenib-induced increase in c-Myc and c-Met expression. HSP90 inhibitors have been explored as potential treatments for various cancers [49, 50]. Our findings also showed that the combination of an HSP90 inhibitor and sorafenib treatment enhanced cytotoxicity compared to monotherapy. Additionally, it is interesting to note that AFP expression and the AFP-HSP90-Myc/Met axis may also influence the effectiveness of cisplatin in gastric cancer cells. Cisplatin is a commonly used chemotherapy drug for advanced gastric cancer [51], and studies have shown that increased expression of c-Myc and c-Met sensitizes gastric cancercells to cisplatin treatment [52, 53]. Our study revealed that AFP-HSP90 inhibition increased the expression of c-Myc and c-Met, while AFP-HSP90 inhibition increased the sensitivity of gastric cancer cells to cisplatin and had an inhibitory effect on the expression of c-Myc and c-Met. These findings suggest that the AFP-HSP90-Myc/Met axis may contribute to chemoresistance in HCC, and targeting this axis, either through AFP knockdown or HSP90 inhibition, could enhance the effectiveness of sorafenib and cisplatin in treating AFP-producing HCC and gastric cancer, respectively.

In conclusion, this study provides insights into the role of AFP in promoting HCC and gastric cancer proliferation through c-Myc and c-Met regulation. The AFP-HSP90-Myc/Met axis emerges as a potential pathway in tumor progression and chemoresistance in AFP-producing tumors. Inhibiting the AFP-HSP90-Myc/Met axis, particularly through HSP90 inhibitors, hold promise for treating AFP-producing tumors, and their combination with sorafenib therapy may enhance therapeutic efficacy. Further studies are needed to assess the safety and effectiveness of this approach in multi-agent chemotherapy, emphasizing AFP as a potential biomarker for the development of targeted cancer therapies.

### Supplementary Information

Additional file 1.

Additional file 2.

Additional file 3.

## Data Availability

We did not upload any of the data generated during the study to external repositories in the public domain. The lead contact for this study will make all data reported in the paper available upon request. Please contact the lead contact if you would like to obtain access to the data.

## Abbreviations

AFP: Alpha-fetoprotein
HCC: Hepatocellular carcinoma
GC: Gastric carcinoma
AFPGC: AFP-producing gastric carcinoma
c-MYC: Cellular-myelocytomatosis viral oncoprotein
c-MET: Cellular-mesenchymal epithelial transition factor
HSP90: Heat shock protein 90

## References

[1] Schieving JH, de Vries M, van Vugt JMG, Weemaes C, van Deuren M, Nicolai J, et al. Alpha-fetoprotein, a fascinating protein and biomarker in neurology. Eur J Paediatr Neurol. 2014;18:243–8. 24120489 10.1016/j.ejpn.2013.09.003

[2] Wang XW, Xie H. Alpha-fetoprotein enhances the proliferation of human hepatoma cells in vitro. Life Sci. 1999;64:17–23. 10027738 10.1016/S0024-3205(98)00529-3

[3] Renaud M, Tranchant C, Koenig M, Anheim M. Autosomal recessive cerebellar ataxias with elevated alpha-fetoprotein: uncommon diseases. Common Biomarker Mov Disord. 2020;35:2139–49. 33044027 10.1002/mds.28307

[4] Cuckle HS, Wald NJ, Lindenbaum RH. Maternal serum alpha-fetoprotein measurement: a screening test for Down syndrome. Lancet. 1984;1:926–9. 6201687 10.1016/S0140-6736(84)92389-4

[5] De Mees C, Laes J-F, Bakker J, Smitz J, Hennuy B, Van Vooren P, et al. Alpha-fetoprotein controls female fertility and prenatal development of the gonadotropin-releasing hormone pathway through an antiestrogenic action. Mol Cell Biol. 2006;26:2012–8. 16479017 10.1128/MCB.26.5.2012-2018.2006 PMC1430253

[6] Gabant P, Forrester L, Nichols J, Van Reeth T, De Mees C, Pajack B, et al. Alpha-fetoprotein, the major fetal serum protein, is not essential for embryonic development but is required for female fertility. Proc Natl Acad Sci U S A. 2002;99:12865–70. 12297623 10.1073/pnas.202215399 PMC130551

[7] Mitsuhashi N, Kobayashi S, Doki T, Kimura F, Shimizu H, Yoshidome H, et al. Clinical significance of alpha-fetoprotein: involvement in proliferation, angiogenesis, and apoptosis of hepatocellular carcinoma. J Gastroenterol Hepatol. 2008;23:e189–97. 18466288 10.1111/j.1440-1746.2008.05340.x

[8] Li P, Wang S-S, Liu H, Li N, McNutt MA, Li G, et al. Elevated serum alpha fetoprotein levels promote pathological progression of hepatocellular carcinoma. World J Gastroenterol. 2011;17:4563–71. 22147961 10.3748/wjg.v17.i41.4563 PMC3226982

[9] Hirajima S, Komatsu S, Ichikawa D, Kubota T, Okamoto K, Shiozaki A, et al. Liver metastasis is the only independent prognostic factor in AFP-producing gastric cancer. World J Gastroenterol. 2013;19:6055–61. 24106406 10.3748/wjg.v19.i36.6055 PMC3785627

[10] Chen J, Röcken C, Treiber G, Jentsch-Ulrich K, Malfertheiner P, Ebert MPA. Clinical implications of alpha-fetoprotein expression in gastric adenocarcinoma. Dig Dis. 2003;21:357–62. 14752227 10.1159/000075360

[11] Li M, Zhou S, Liu X, Li P, McNutt MA, Li G. alpha-Fetoprotein shields hepatocellular carcinoma cells from apoptosis induced by tumor necrosis factor-related apoptosis-inducing ligand. Cancer Lett. 2007;249:227–34. 17046153 10.1016/j.canlet.2006.09.004

[12] Li M, Liu X, Zhou S, Li P, Li G. Effects of alpha fetoprotein on escape of Bel 7402 cells from attack of lymphocytes. BMC Cancer. 2005;5:96. 16080799 10.1186/1471-2407-5-96 PMC1198224

[13] Wang S, Zhu M, Wang Q, Hou Y, Li L, Weng H, et al. Alpha-fetoprotein inhibits autophagy to promote malignant behaviour in hepatocellular carcinoma cells by activating PI3K/AKT/mTOR signalling. Cell Death Dis. 2018;9:1027. 30301886 10.1038/s41419-018-1036-5 PMC6177398

[14] Chen T, Dai X, Dai J, Ding C, Zhang Z, Lin Z, et al. AFP promotes HCC progression by suppressing the HuR-mediated Fas/FADD apoptotic pathway. Cell Death Dis. 2020;11:822. 33009373 10.1038/s41419-020-03030-7 PMC7532541

[15] Li C, Wang S, Jiang W, Li H, Liu Z, Zhang C, et al. Impact of intracellular alpha fetoprotein on retinoic acid receptors-mediated expression of GADD153 in human hepatoma cell lines. Int J Cancer. 2012;130:754–64. 21365646 10.1002/ijc.26025

[16] Wang S, Jiang W, Chen X, Zhang C, Li H, Hou W, et al. Alpha-fetoprotein acts as a novel signal molecule and mediates transcription of Fn14 in human hepatocellular carcinoma. J Hepatol. 2012;57:322–9. 22521346 10.1016/j.jhep.2012.03.029

[17] Zhang C, Chen X, Liu H, Li H, Jiang W, Hou W, et al. Alpha fetoprotein mediates HBx induced carcinogenesis in the hepatocyte cytoplasm. Int J Cancer. 2015;137:1818–29. 25846475 10.1002/ijc.29548

[18] Parpart S, Roessler S, Dong F, Rao V, Takai A, Ji J, et al. Modulation of miR-29 expression by α-fetoprotein is linked to the hepatocellular carcinoma epigenome. Hepatology. 2014;60:872–83. 24798303 10.1002/hep.27200 PMC4146718

[19] Stine ZE, Walton ZE, Altman BJ, Hsieh AL, Dang CV. MYC, metabolism, and cancer. Cancer Discov. 2015;5:1024–39. 26382145 10.1158/2159-8290.CD-15-0507 PMC4592441

[20] Liu P, Ge M, Hu J, Li X, Che L, Sun K, et al. A functional mammalian target of rapamycin complex 1 signaling is indispensable for c-Myc-driven hepatocarcinogenesis. Hepatology. 2017;66:167–81. 28370287 10.1002/hep.29183 PMC5481473

[21] Dang CV. MYC on the path to cancer. Cell. 2012;149:22–35. 22464321 10.1016/j.cell.2012.03.003 PMC3345192

[22] Anauate AC, Leal MF, Calcagno DQ, Gigek CO, Karia BTR, Wisnieski F, et al. The complex network between MYC oncogene and microRNAs in gastric cancer: an overview. Int J Mol Sci. 2020;21:1782. 32150871 10.3390/ijms21051782 PMC7084225

[23] Dang CV. c-Myc target genes involved in cell growth, apoptosis, and metabolism. Mol Cell Biol. 1999;19. 10.1128/mcb.19.1.1 PMC83860 9858526

[24] Gherardi E, Birchmeier W, Birchmeier C, Vande Woude G. Targeting MET in cancer: rationale and progress. Nat Rev Cancer. 2012;12:89. 22270953 10.1038/nrc3205

[25] Cecchi F, Rabe DC, Bottaro DP. Targeting the HGF/Met signalling pathway in cancer. Eur J Cancer. 2010;46:1260–70. 20303741 10.1016/j.ejca.2010.02.028 PMC3412517

[26] Han H, Jain AD, Truica MI, Izquierdo-Ferrer J, Anker JF, Lysy B, et al. Small-molecule MYC inhibitors suppress tumor growth and enhance immunotherapy. Cancer Cell. 2019;36:483. 31679823 10.1016/j.ccell.2019.10.001 PMC6939458

[27] Dejure FR, Royla N, Herold S, Kalb J, Walz S, Ade CP, et al. The MYC mRNA 3′-UTR couples RNA polymerase II function to glutamine and ribonucleotide levels. EMBO J. 2017;36:1854–68. 28408437 10.15252/embj.201796662 PMC5494468

[28] Guan L, Zou Q, Liu Q, Lin Y, Chen S. HSP90 inhibitor ganetespib (STA-9090) inhibits tumor growth in c-Myc-dependent esophageal squamous cell carcinoma. Onco Targets Ther. 2020;13:2997–3011. 32308431 10.2147/OTT.S245813 PMC7156265

[29] Wang S, Pashtan I, Tsutsumi S, Xu W, Neckers L. Cancer cells harboring MET gene amplification activate alternative signaling pathways to escape MET inhibition but remain sensitive to Hsp90 inhibitors. Cell Cycle. 2009;8:2050–6. 19502802 10.4161/cc.8.13.8861 PMC7282701

[30] Taipale M, Krykbaeva I, Koeva M, Kayatekin C, Westover KD, Karras GI, et al. Quantitative analysis of Hsp90-client interactions reveals principles of substrate recognition. Cell. 2012;150:987–1001. 22939624 10.1016/j.cell.2012.06.047 PMC3894786

[31] Llovet JM, Peña CEA, Lathia CD, Shan M, Meinhardt G, Bruix J. Plasma biomarkers as predictors of outcome in patients with advanced hepatocellular carcinoma. Clin Cancer Res. 2012;18:2290–300. 22374331 10.1158/1078-0432.CCR-11-2175 PMC12268944

[32] Forner A, Reig M, Bruix J. Hepatocellular carcinoma. The Lancet. 2018;391:1301–14. 10.1016/S0140-6736(18)30010-2 29307467

[33] Gabay M, Li Y, Felsher DW. MYC activation is a hallmark of cancer initiation and maintenance. Cold Spring Harb Perspect Med. 2014;4. 10.1101/cshperspect.a014241 PMC4031954 24890832

[34] Nouri-Vaskeh M, Alizadeh L, Hajiasgharzadeh K, Mokhtarzadeh A, Halimi M, Baradaran B. The role of HSP90 molecular chaperones in hepatocellular carcinoma. J Cell Physiol. 2020;235:9110–20. 32452023 10.1002/jcp.29776

[35] Xie M, Yu T, Jing X, Ma L, Fan Y, Yang F, et al. Exosomal circSHKBP1 promotes gastric cancer progression via regulating the miR-582-3p/HUR/VEGF axis and suppressing HSP90 degradation. Mol Cancer. 2020;19:112. 32600329 10.1186/s12943-020-01208-3 PMC7322843

[36] Xu Q, Tu J, Dou C, Zhang J, Yang L, Liu X, et al. HSP90 promotes cell glycolysis, proliferation and inhibits apoptosis by regulating PKM2 abundance via Thr-328 phosphorylation in hepatocellular carcinoma. Mol Cancer. 2017;16:178. 29262861 10.1186/s12943-017-0748-y PMC5738801

[37] Wu J, Liu T, Rios Z, Mei Q, Lin X, Cao S. Heat shock proteins and cancer. Trends Pharmacol Sci. 2017;38:226–56. 28012700 10.1016/j.tips.2016.11.009

[38] Barrott JJ, Haystead TAJ. Hsp90, an unlikely ally in the war on cancer. FEBS J. 2013;280:1381–96. 23356585 10.1111/febs.12147 PMC3815692

[39] Augello G, Emma MR, Cusimano A, Azzolina A, Mongiovì S, Puleio R, et al. Targeting HSP90 with the small molecule inhibitor AUY922 (luminespib) as a treatment strategy against hepatocellular carcinoma. Int J Cancer. 2019;144:2613–24. 30488605 10.1002/ijc.31963

[40] Liang X-Q, Li K-Z, Li Z, Xie M-Z, Tang Y-P, Du J-B, et al. Diagnostic and prognostic value of plasma heat shock protein 90alpha in gastric cancer. Int Immunopharmacol. 2021;90: 107145. 33162344 10.1016/j.intimp.2020.107145

[41] Wei W, Liu M, Ning S, Wei J, Zhong J, Li J, et al. Diagnostic value of plasma HSP90α levels for detection of hepatocellular carcinoma. BMC Cancer. 2020;20:6. 31898536 10.1186/s12885-019-6489-0 PMC6941289

[42] Miyajima N, Tsutsumi S, Sourbier C, Beebe K, Mollapour M, Rivas C, et al. The HSP90 inhibitor ganetespib synergizes with the MET kinase inhibitor crizotinib in both crizotinib-sensitive and -resistant MET-driven tumor models. Cancer Res. 2013;73:7022–33. 24121490 10.1158/0008-5472.CAN-13-1156 PMC7561255

[43] Khandelwal A, Crowley VM, Blagg BSJ. Natural product inspired N-terminal Hsp90 inhibitors: from bench to bedside? Med Res Rev. 2016;36:92. 26010985 10.1002/med.21351 PMC4659773

[44] Song S, Bai M, Li X, Gong S, Yang W, Lei C, et al. Early predictive value of circulating biomarkers for sorafenib in advanced hepatocellular carcinoma. Expert Rev Mol Diagn. 2022;22:361–78. 35234564 10.1080/14737159.2022.2049248

[45] Firtina Karagonlar Z, Koc D, Iscan E, Erdal E, Atabey N. Elevated hepatocyte growth factor expression as an autocrine c-Met activation mechanism in acquired resistance to sorafenib in hepatocellular carcinoma cells. Cancer Sci. 2016;107:407–16. 26790028 10.1111/cas.12891 PMC4832867

[46] Chen J, Jin R, Zhao J, Liu J, Ying H, Yan H, et al. Potential molecular, cellular and microenvironmental mechanism of sorafenib resistance in hepatocellular carcinoma. Cancer Lett. 2015;367:1–11. 26170167 10.1016/j.canlet.2015.06.019

[47] Xia P, Zhang H, Xu K, Jiang X, Gao M, Wang G, et al. MYC-targeted WDR4 promotes proliferation, metastasis, and sorafenib resistance by inducing CCNB1 translation in hepatocellular carcinoma. Cell Death Dis. 2021;12:691. 34244479 10.1038/s41419-021-03973-5 PMC8270967

[48] Wang J-W, Ma L, Liang Y, Yang X-J, Wei S, Peng H, et al. RCN1 induces sorafenib resistance and malignancy in hepatocellular carcinoma by activating c-MYC signaling via the IRE1α–XBP1s pathway. Cell Death Discov. 2021;7:298. 34663798 10.1038/s41420-021-00696-6 PMC8523720

[49] Goyal L, Wadlow RC, Blaszkowsky LS, Wolpin BM, Abrams TA, McCleary NJ, et al. A phase I and pharmacokinetic study of ganetespib (STA-9090) in advanced hepatocellular carcinoma. Invest New Drugs. 2015;33:128. 25248753 10.1007/s10637-014-0164-8

[50] Kawazoe A, Itahashi K, Yamamoto N, Kotani D, Kuboki Y, Taniguchi H, et al. TAS-116 (Pimitespib), an Oral HSP90 inhibitor, in combination with nivolumab in patients with colorectal cancer and other solid tumors: an open-label, dose-finding, and expansion phase Ib trial (EPOC1704). Clin Cancer Res. 2021;27:6709–15. 34593531 10.1158/1078-0432.CCR-21-1929

[51] Mahlberg R, Lorenzen S, Thuss-Patience P, Heinemann V, Pfeiffer P, Möhler M. New perspectives in the treatment of advanced gastric cancer: S-1 as a novel oral 5-FU therapy in combination with cisplatin. Chemotherapy. 2017;62:62–70. 27643822 10.1159/000443984

[52] Yang X, Cai H, Liang Y, Chen L, Wang X, Si R, et al. Inhibition of c-Myc by let-7b mimic reverses mutidrug resistance in gastric cancer cells. Oncol Rep. 2015;33:1723–30. 25633261 10.3892/or.2015.3757

[53] Zhang Q, Zhang H, Ning T, Liu D, Deng T, Liu R, et al. Exosome-delivered c-Met siRNA could reverse chemoresistance to cisplatin in gastric cancer. Int J Nanomedicine. 2020;15:2323–35. 32308384 10.2147/IJN.S231214 PMC7133545

