# Supplementary figures and images for: AFP-HSP90 mediated MYC/MET activation promotes tumor progression in hepatocellular carcinoma and gastric cancers
Source: Cancer Cell Int. 2024 Aug 12;24:283. doi: 10.1186/s12935-024-03455-6 (PMC11321088; doi:10.1186/s12935-024-03455-6)

**Figure. S1**

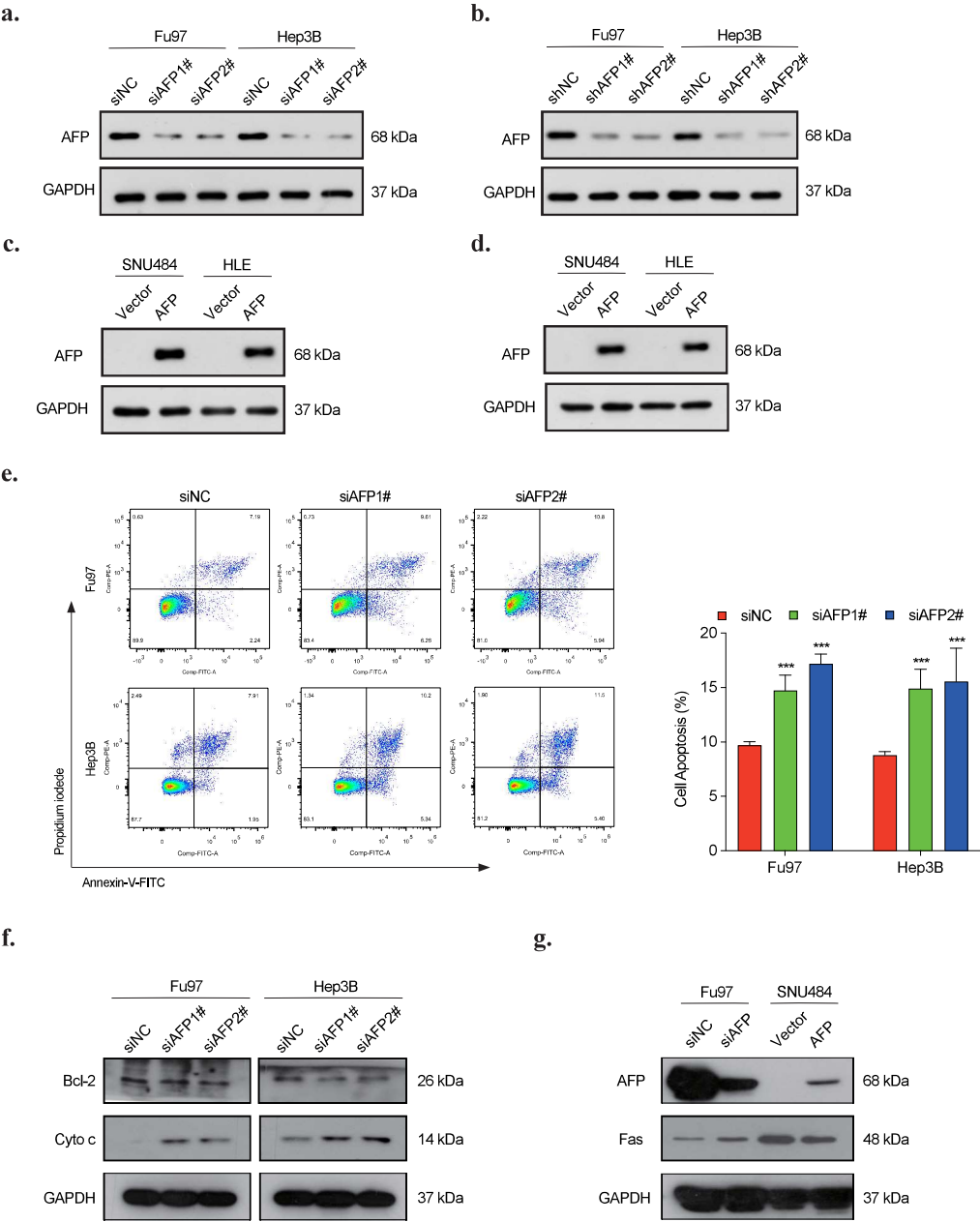

Figure. S2

a.

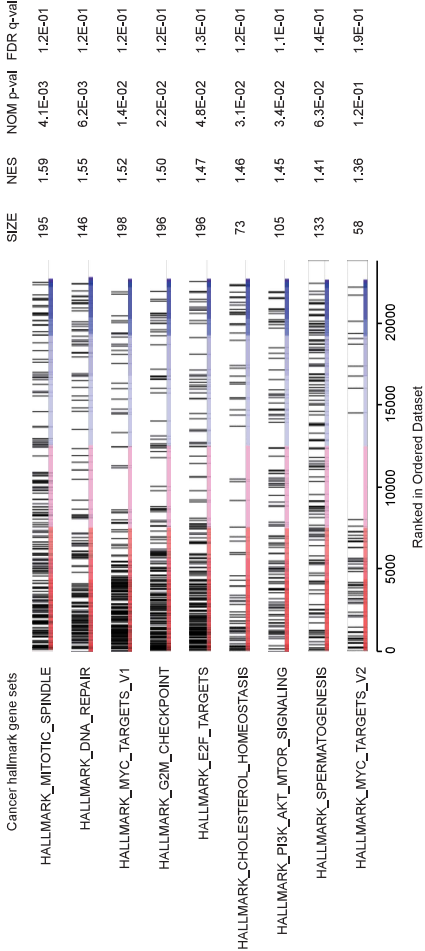

b.

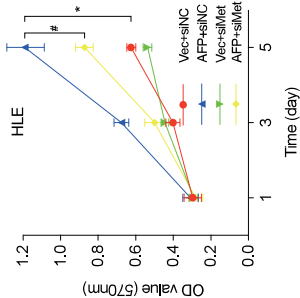

c.

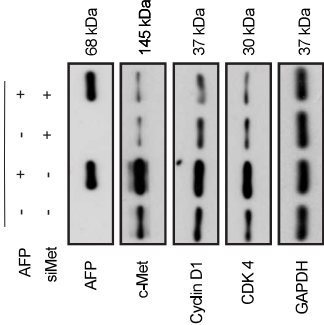

**Figure. S3**

**a.**

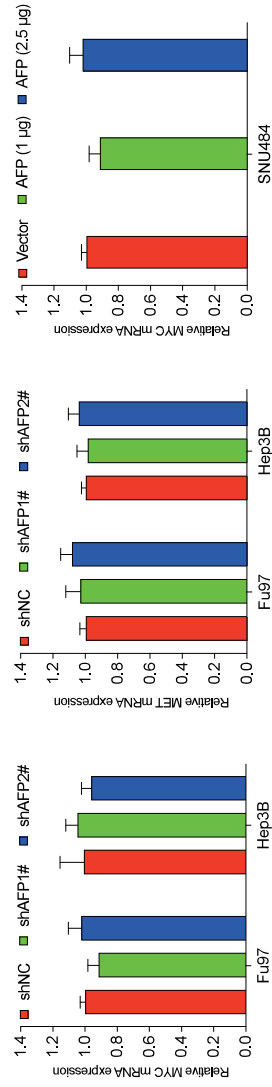

**b.**

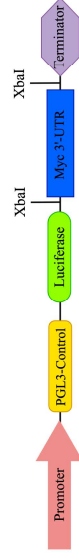

**c.**

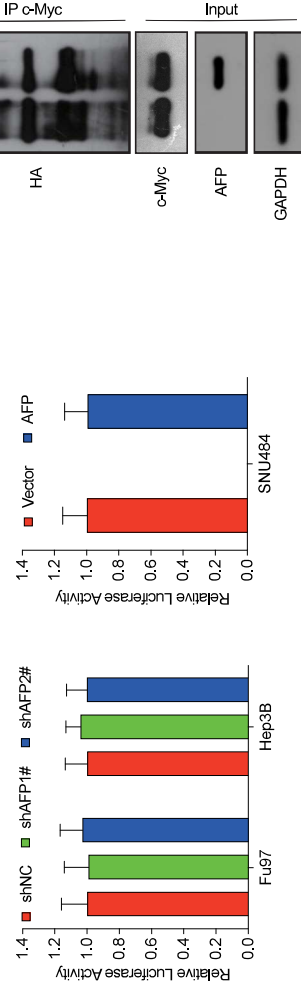

**Figure. S4**

a.

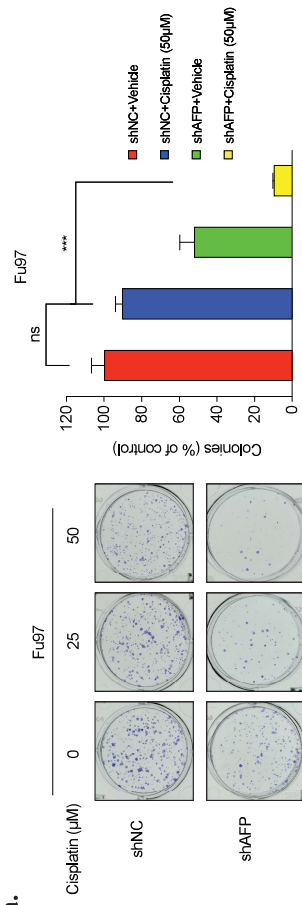

b.

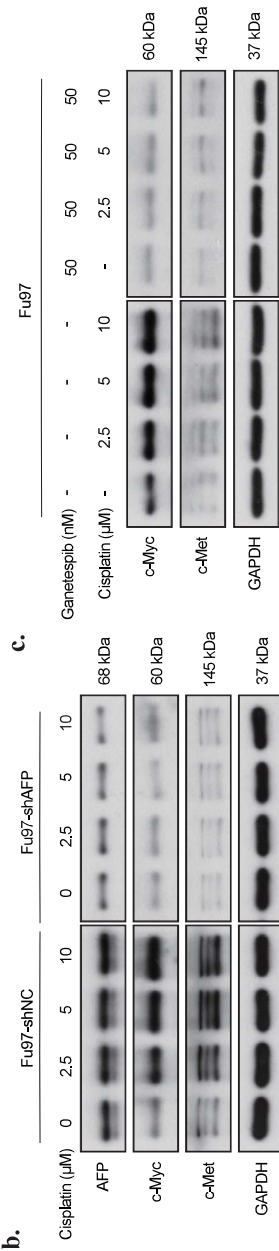

Supplement: Supplementary file 1 — Additional file 1. [file 12935_2024_3455_MOESM1_ESM.pdf]
