# Supplementary material for: AFP-HSP90 mediated MYC/MET activation promotes tumor progression in hepatocellular carcinoma and gastric cancers
Source: Cancer Cell Int. 2024 Aug 12;24:283. doi: 10.1186/s12935-024-03455-6 (PMC11321088; doi:10.1186/s12935-024-03455-6)
Supplement: Supplementary file 2 — Additional file 2. [file 12935_2024_3455_MOESM2_ESM.docx]

**Title:** **AFP-HSP90 mediated MYC/MET activation promotes tumor progression in hepatocellular carcinoma and gastric cancers**

**Supplementary Figure Legends**

**Figure S1.**

(a) Expression of AFP at the protein level in Fu97 and Hep3B cells transiently transfected with siNC and AFP siRNAs.

(b) Expression of AFP at the protein level in Fu97 and Hep3B cells stably infected with shNC and AFP shRNAs

(c) Expression of AFP at the protein level in SNU484 and HLE cells transiently transfected with a control vector and an AFP plasmid.

(d) Expression of AFP at the protein level in SNU484 and HLE cells stably transfected with a control vector and an AFP plasmid.

(e) Flow cytometry analysis with Annexin V- FITC/PI staining was performed on Fu97 and Hep3B cells stably expressing AFP/control shRNAs. The percentage of apoptosis cells was calculated, and the experiments were performed in triplicates. Data are presented as the mean ± SE. *p < 0.05 versus siNC.

(f) Indicated cells were transfected with negative control or AFP siRNAs. Cell lysates were immunoblotted and analyzed with Bcl-2, Bax and Cyto c antibodies.

(g) Indicated cells were transfected with AFP siRNA or AFP plasmid. Cell lysates were immunoblotted and analyzed with AFP and Fas antibodies.

**Figure S2.**

(a) GSEA normalized enrichment scores (NES), normalized p value and FDR-q value for upregulated hallmark gene sets are indicated.

(b) Cell proliferation assay was performed in HLE cells transfected with control plasmid, AFP plasmid, c-Met siRNA, c-Met siRNA and AFP plasmid (n = 5). *p < 0.05 vs Vec + siNC, #P< 0.05 vs AFP + siMet.

(c) HLE cells were transfected with AFP plasmid or c-Met siRNAs or negative control. Cell lysates were immunoblotted and analyzed with indicated antibodies.

**Figure S3.**

(a) qRT-PCR analysis of MYC mRNA levels (left panel) and MET mRNA levels (middle panel) following AFP knockdown in Fu97 or Hep3B cells. (right panel)

(b) Effect of AFP knockdown on the activity of a luciferase reporter bearing the human c-Myc 3′-UTR region. A schematic representation of the luciferase reporter constructs with the human c-Myc 3′-UTR is shown (top). A luciferase reporter construct was cotransfected into Fu97 and Hep3B cells transfected with AFP siRNAs (siAFP1# and siAFP 2#) or a negative control (siNC), and HLE cells transfected with AFP plasmid or control vector.

(c) AFP inhibits c-Myc ubiquitination. SNU484 cells were transfected with AFP and HA-Ub and indicated plasmids, following MG132 treatment (25μM) for 6 h. Ubiquitinated c-Myc was purified by anti-Myc antibody and detected by immunoblotting using anti-HA antibody.

**Figure S4.**

(a) Colony formation assays of AFP knockdown Fu97 cells treated with cisplatin. Fu97 (10000 cells per well) infected with lentivirus containing control shRNA (shNC) or shRNA targeting AFP (shAFP) were incubated with indicated concentration of cisplatin for two weeks, and the cells were fixed with methanol and stained with crystal violet. Experiments were performed in triplicates.

(b) Western blot assays were performed in AFP knockdown Fu97 (Fu97-shAFP) and control cells (Fu97-shNC) treated with indicated concentration of cisplatin. AFP, c-Myc and c-Met expression were detected with indicated antibody.

(c) Western blot assays were performed in Fu97 cells treated with indicated concentration of cisplatin and ganetespib. c-Myc and c-Met expression were detected with indicated antibody.
