# Supplementary material for: AFP-HSP90 mediated MYC/MET activation promotes tumor progression in hepatocellular carcinoma and gastric cancers
Source: Cancer Cell Int. 2024 Aug 12;24:283. doi: 10.1186/s12935-024-03455-6 (PMC11321088; doi:10.1186/s12935-024-03455-6)
Supplement: Supplementary file 3 — Additional file 3. [file 12935_2024_3455_MOESM3_ESM.docx]

**Table S1.** Sequences used for siRNA and shRNA interference.

| siRNA/shRNA | Sequence（5' to 3'） |
| --- | --- |
| siAFP/siAFP 1# | AUAAGUGUCCGAUAAUAAUGUCAGC |
| siAFP 2# | CCAGAACACUGCAUAGAAATT |
| shAFP/shAFP 1# | AUAAGUGUCCGAUAAUAAUGUCAGC |
| shAFP 2# | CCAGAACACUGCAUAGAAATT |
| si-Myc | GAGGAUAUCUGGAAGAAAUTT |
| si-Met | AACATGGCTCTAGTTGTCGAC |

**Table S2.** Primers used for qRT-PCR.

| Primers | Sequence（5' to 3'） |
| --- | --- |
| qPCR-AFP-forward | CCAACAGGAGCCATGCTT |
| qPCR-AFP-reverse | GAATGCAGGAGGGACATATGTTT |
| qPCR-MYC-forward | AGCGACTCTGAGGAGGAACAA |
| qPCR-MYC-reverse | GTGGGCTGTGAGGAGGTTTG |
| qPCR-MET-forward | CACTTCTGAGAAATTCATCAGGCTGTGAAG |
| qPCR-MET-reverse | AGAGGACTTCGCTGAATTGACCCATG |
| qPCR-GAPDH-forward | ACGGATTTGGTCGTATTGGGC |
| qPCR-GAPDH-reverse | CTCGCTCCTGGAAGATGGTGAT |
